# Salivary gland choristoma in the buccinator muscle: A case report and literature review

**DOI:** 10.4317/jced.52391

**Published:** 2015-10-01

**Authors:** Dídac Sotorra-Figuerola, Nieves Almendros-Marqués, Antonio-Jesús Espana-Tost, Eduard Valmaseda-Castellón, Cosme Gay-Escoda

**Affiliations:** 1 DDS. Fellow of the Master of Oral Surgery and Implantology, School of Dentistry, University of Barcelona. Barcelona, Spain; 2 DDS, MS. Associate Professor of Oral Surgery and Professor of Master of Oral Surgery and Implantology, School of Dentistry, University of Barcelona. Barcelona, Spain; 3 MD, DDS, MS, PhD. Associate Professor of Oral Surgery and Professor of Master of Oral Surgery and Implantology. Chairman of European Master Degree in Oral Laser Applications (EMDOLA). School of Dentistry, University of Barcelona. Investigator of the IDIBELL institute. Barcelona, Spain; 4 DDS, MS, PhD. Professor of Oral Surgery. Director of Master of Oral Surgery and Implantology, School of Dentistry, University of Barcelona. Investigator of the IDIBELL institute. Barcelona, Spain; 5 MD, DDS, MS, PhD. Chairman and Professor of Oral and Maxillofacial Surgery. Faculty of Dentistry, University of Barcelona. Director of the Master of Oral Surgery and Implantology (EFHRE International University/FUCSO). Coordinating investigator of the IDIBELL institute. Head of the Department of Oral and Maxillofacial Surgery and Implantology, and the Director of the TMJ Disease and Orofacial Pain Unit. Teknon Medical Center. Barcelona, Spain

## Abstract

Salivary gland choristoma (SGCh) is defined as the presence of normal salivary tissue in an abnormal location. It is a rare entity in oral and maxillofacial region and its ethiology is unknown. The typical presentation of salivary gland heterotopia is an asymptomatic mass that may or may not produce saliva. Some examples of ectopic salivary tissue in the pituitary gland, in the lymph nodes, in the middle ear, in the neck, in the jaw, in the thyroid gland, in the mediastinum and in the rectum have been documented in literature. We report the case of a 61-year-old male presented with a bilateral tumorlike mass in the cheek. The mass was painless, of fibrous consistency and had size change with time. The histological diagnosis was salivary gland choristoma in the buccinator muscle. In this article, we will revise the characteristic of salivary gland heterotopias and we present a report case that has not been described in literature: a bilateral choristoma of salivary gland in the buccinator muscle, which should be included in the differential diagnosis of head and neck masses.

** Key words:**Choristoma, heterotopias, ectopic tissue, salivary gland, buccinator muscle.

## Introduction

Salivary gland choristoma (SGCh) is defined as the presence of normal salivary tissue that appears in an ectopic location. It is also denominated as *aberrant salival tissue, salival heterotopia and ectopic salival tissue* (1). It is different from hamartoma because the latter appears in normal location.

SGCh has been documented in several areas of the body but rarely in the orofacial region (1,2). The most common locations are the posterior lobe of pituitary gland, the periparotid lymph nodes, the middle ear and the lower neck. Less common areas include upper neck, lingual mandible, external auditory canal, thyroid gland, mediastinum, prostate gland, vulva and rectum (3).

Several different heterotopias as osseous choristoma, cartilaginous choristoma, gastric mucosal choristoma, glial choristoma and thyroid choristoma have been described in oral and maxillofacial region (4).

The etiopathogenesis of this entity remains uncertain although it is believed that it is due to a developmental anomaly (5). Sex and age predilection have not been described and the most typical clinical presentation, although it depends from choristoma location, is an asymptomatic mass. However, SGCh can segregate saliva when stimulated (6).

The present case is the first described in the literature of a salivary gland choristoma in the buccinator muscle.

## Report of Case

A 61-year-old man was referred to the Unit of Oral Surgery of the University of Barcelona in January of 2010 presenting a bilateral tumefaction in the genian region. The patient had a chronic gastritis and reflux gastritis treated by metoclopramide since 1990 and a C-reactive protein level increase in his last blood analysis. He had seen treated of a depression since 1995. He didn’t have either allergies or toxic habits. He had undergone amigdalectomy (1967) and appendicectomy (1972).

The patient had a bilateral mass in both cheeks, rounded, 1.5 cm of diameter, painless, of fibrous consistency, adhering to deep structures, without erythema and with a reported evolution of 2 years. The most striking clinical feature was sporadic swelling, associated sometimes with food intake. The swelling developed in 5-7 days and remained from 15 days to some months. The patient visited an Oral and Maxillofacial Surgery service a year ago and was treated with antihistaminics and muscular relaxants that were unsuccessful to solve his complain.

**Figure 1:**
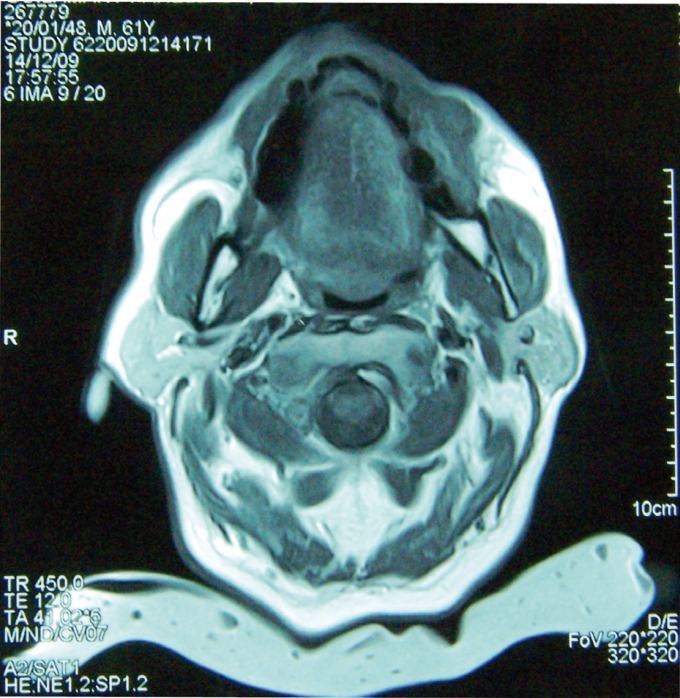
Magnetic Resonance. Axial plane was informed as unspecific pathology of the buccinator muscle.

A magnetic resonance of middle and lower face with axial planes was informed as unspecific pathology of the buccinator muscle (Fig. 1).

**Figure 2:**
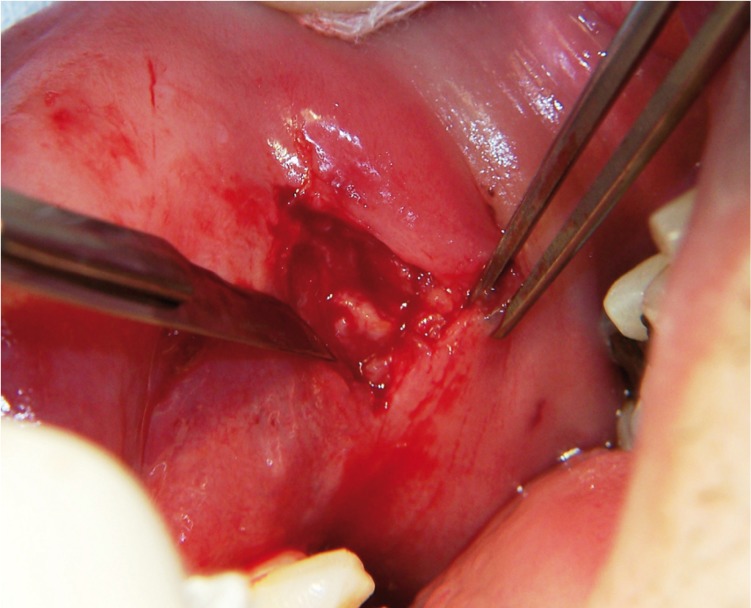
Dissection of the left side lesion.

An incisional biopsy was perfomed with local anesthesia and intraoral approach. The incision was 2 cm in length and reached the buccinator muscle where the lesion was located, and two samples of 0.5 cm of diameter were taken (Fig. 2).

**Figure 3:**
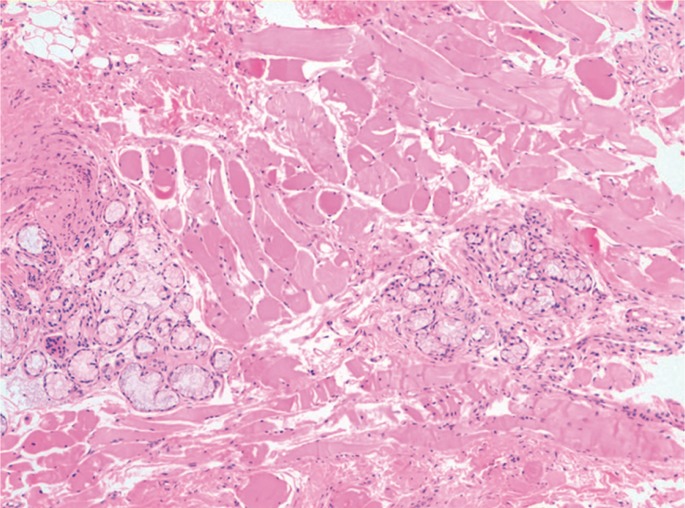
Histologic image of the removed tissue (hematoxylin and eosin stain). It shows skeletal muscle with normal salivary gland acinis inside and unspecific chronic inflammation. Magnifications: 40X. *Courtesy of Dr. August Vidal.*

The histopathologic examination of the removed tissue described the presence of buccinator muscle fibres mixed with normal salivary gland acinis (choristoma of salivary gland), fibrosis and unspecific chronic inflammation (Fig. 3).

Three months after the operation the right cheek experienced some swelling (approximately 1 cm increase in diameter). We explained to the patient the benign nature of the lesion and he decided not to have it removed.

## Discussion

The criteria for diagnosis of choristoma are a tumorlike growth in the soft tissues or an osseous radiolucency in a radiograph, histologically an ectopic tissue with a normal pattern, with no neoplastic features and topographically a tissue’s type not normally found in the region (4). SGCh has been documented in several areas of the organism, being the posterior lobe of pituitary gland, the parathyroid gland, the middle ear, the lymph nodes and the lower neck the most common locations. With less common fre-quency, there are case reports of the upper neck, the lingual mandible, the thyroid gland, the mediastinum, the optic nerve, the bronchus, the prostate gland and the rectum were published (3,6-10). This case report is exceptional, it isn’t any SGCh in the buc-cinator muscle, although a SGCh that affected the mandible and masseter muscle was described (11).

In literature, it has been reported that the age of diagnosis of this entity is variable, between birth to 81 years old (4,8,12,13). However, Haemel *et al.* explain that many lesions in this series were noted at birth or early in childhood and the late diagnosis were when the size was very small or there was an absence of symptoms is nonexistent (6). There is not any evidence about sex preference (7,12,13).

The presentation of bilateral and symmetric SGCh, just as our case, is very rare. However, few cases of bilateral choristomas in the neck have been described in literature (6,7).

The most frequent clinical presentation is a painless tumor (7). This mass may be asymptomatic or present an intermittent production of a clear fluid as saliva associated with eating. Inflammatory signs can occur (1,6,7). The present patient presented periods of swelling of the cheeks associated with copious eating and but there was no evidence drainage.

It must be taken into account that the clinical presentation depends of location. SGCh in the jaw, called Stafne bone cavity, are usually radiological findings (4,14). Nevertheless, SGCh in the middle ear may cause hypoacusia of conduction, disformation of the internal auditory canal and facial paralysis (13,15) and salivary gland ectopia in the rectum may manifest with rectal bleeding and/or tenesmus (7).

Aetiopathogenesis is unknown but it is believed to be related to embrionary development (1,2,4,5,13). In 1968, Willis suggested 3 general hypotheses: a) abnormal persistence and development of vestigial structures; b) the abnormal differentiation of local tissues; and c) the dislocation of a portion of a definitive rudimentary organ during its movement and development (2,8). Although, Hsu *et al.* (16) reported a case of a family with five generation suffering salivary gland heterotopias in the neck suggesting the existence of an autosomal dominant inheritance, some authors explain the choristoma’s origin as a type of metaplasya or development disorder where undifferentiated stem cells become mature salivary tissue (12).

The SGCh histology shows normal salival tissue, mucous and mixed acini predilection that may be accompanied with fibrosis and, sometimes, may difficult the histological diagnose. Some of the variation seen within the associated ducts, such as areas of squamous metaplasia, may be related to chronic inflammation and lymph infiltration secondary to physical pressure of retained mucus (6,7). The present case showed these histological features and the changes in size were probably due to mucosal secretion and chronic swelling.

Tumours in SGCh are rare and 80% of them are benign (6,8). The most frequent benign neoplasm is Whartin’s tumour followed by pleomorphic adenoma. The most described malignant neoplasm is mucoepidermoid carcinoma (8). In the diagnosis of a ma-lignant neoplasm of salival aberrant tissue, it is important to consider the possibility of it being a metastasis of a primary salivary gland tumour (2,6,8).

There is much controversy in the treatment of SGCh because of the malignization possibility. There are two opinions. In one hand, some authors believe that salival heterotopia is a normal tissue and doesn’t require complete excision when it is diagnosed histologically. Choristoma removal is only necessary when there are signs of infection or neoplasm (1,13,17). In the other hand, some authors prefer the complete removal of the mass (1,2,6,12,18). Although malignization is very rare, choristomas are imma-ture in nature, which theoretically increase the chancy to malignization (19).
